# Cholera—What Is It?

**Published:** 1883-08-20

**Authors:** T. S. Bell


### CHOLERA. WHAT IS IT?

By Prof. T. S. Bell, M.D.

This potential word exercises a very strange power over the human mind. Nations stand affrighted at the utterance of the word. The lessons of experience are forgotten, the indubitable facts upon which all truth stands become nugatory in the frenzied excitement of the mind, and people fail to treasure up observations that would prove valuable if brought into action at the proper time. To recall the lessons of history, to teach the records of nature, to show that when she is heeded her revelations are always beneficent, and when neglected that she exacts a fearful penalty, is the object of this paper. There can be no motive to utter aught but the truth, the whole truth and nothing but the truth. He that is indifferent to these requirements is unworthy of any place in society or any position in the ranks of the medical profession. The office of that profession is to allay unnecessary fears, to calm the public mind, and to conduct it into avenues of safety and to preserve it in health.

Cholera is always a manifestation of a local condition inimical to health and safety. It never showed itself in any spot on the face of the earth except in obedience to this local condition, and wherever the condition exists, no matter where, the disease will infallibly manifest itself. Why did cholera manifest itself in the army of the Marquis of Hastings in 1817? Because it was encamped on the marshy banks of Scind, in tropical weather, where vegetable decomposition was in a fit condition to produce the fatal poison. The pestilence was fearful in that portion of the army near the focal point; that portion of the army encamped away from the river was perfectly healthy, and remained in that condition. The army was ordered to leave the seat of pestilence, and was marched to the hills, where the pestilence ceased. It could be tracked by the skeletons it dropped in its journey to the hills. When the army was in reach of the cause the pestilence raged fearfully; when it reached the hills, there being no cause for pestilence there, sickness ceased, thus vindicating the fact that the intelligent authorities knew the nature of that cause, which, when present, caused the havoc, which, when modified, modified the disease, and which, when absent, prevented its ravages. If, with the eyes of our reason, we were to survey all such outbreaks, we should invariably find the local circumstances that cause them.

Dr. Farr, the Registrar-General of England, looked upon the developments of the disease over a wide-spread area. He looked with the eyes of a philosophic master. He observed with the desire to find the truth, and he endeavored to proclaim that truth with all the clearness in which it presented itself to him. He said, "Cholera is a health inspector whose decrees are infallible, whose requirements are inexorable."

Professor Charles Caldwell, whose medical sagacity was often almost intuitive, said, "Cholera, though a fatal scourge to the world, will, through the wise and beneficent dispensation under which we live, be productive of consequences favorable alike to science and humanity. Besides being instrumental in throwing much light on the practice of physic, it will prove highly influential in extinguishing the belief in pestilential contagion and bringing into disrepute the quarantine and sanitary establishments that have hitherto existed."

There are occasionally gleams of hope in the utterances of leading memhers of the medical profession that foolish chimeras, jejune statements and inanities on the great scourge of the world will cease their warfare against reason, fact and truth, and permit a ripened, clear and matured judgment to exercise its sway over this vital matter of public concern.

In 1848 the British and Foreign Medical Review contained an able and philosophical examination of an immense number of facts connected with the features of cholera, as displayed over a vast extent of the earth's surface. After reviewing these facts the writer closes with these consolatory reflections: "The true philosophy of medicine is the knowledge of the causes of disease, or, if these causes be too subtle and refined for our gross senses, it is the knowledge of the several cenditions, external or internal to the body, which give those causes power. In the future history of medicine we shall see men returning to the principles promulgated by its earliest founders. They will perceive that the treatment of the fully formed disease is, at the same time, the most difficult and the least useful part of this noble profession. They will learn to arrest the evil at the fountain-head, and not to dam the current swollen by a thousand tributaries. And if the principles which we have analyzed in this article be correct, it will not be the least triumph of this philosophy that it has indicated the true mode in which the great epidemic of our time can be most easily and most eflectually controlled. It bars out the disease, not with quarantines and cordon sanitaires, but with a cleanly people and uncontaminated air. *The evil which springs from the bosom of Nature only needs for its removal an observance of the rules which Nature herself reveals.*" The italics are ours.

There are a vast number of prelections upon the contaminations of drinking-water as the cause of enteric fever, contaminations whieh even the mighty powers of the earth cannot remove, according to the assertions of some of the whimsical philosophers who advocate the guess-work involved. But if drinking-water thus becomes contaminated, why may not the air become impure by admixtures of noxious agents? We know that these noxious agents do thus act, because we have many records of men who undertook to plow fields in hot weather—and slept in these fields —every one of whom was found dead next morning. Occurrences similar to this have often taken place in the Campagni di Roma. In that region of verdure called the Maremme di Lucca, in Tuscany, extending from Florence to the shores of the Mediterranean, we know that for centuries the inhabitants never lived at home from the first of July until October, sometimes November, with their frosts put an end to the reign of the poison. The evil has ceased, through the control exercised by an exact knowledge of the character of the conditions from which the poison derived its potency. We shall recur to this again while on this subject.

I am often asked whether certain cases of cholera are instances of the Asiatic variety. I answer, no; they are home-productions, precisely as cases of intermittent fever are. Asia produces intermittent fever analogous to our forms of the fever. The cases which occur in Asia are Asiatic; those that take place in this country are home-productions, or cases of American intermittent fever. The two forms are precisely alike in origin, paroxysms and termination. In a similar way those cases of cholera that occur in Asia are Asiatic; those which we have in America are cases of American cholera. The features of the one are alike in everything to those in the other. In Asia some cases die in an hour after the first symptom shows itself; such cases occur here. In Asia many fatal cases have neither vomiting nor purging; many such cases have been seen here. In all attacks of cholera in Asia there is a total suppression of every secretion, of every nutritive force, of everything like the circulation of the blood· These are equivalent to death. Precisely similar phenomena are found in every endemic of cholera in Louisville. In these conditions of the forces of animal life, in the cases that appear in Asia, the brain towers aloft serenely, and is often mischievous in its trickery. It is often astonishing, when the flitting shadows of life are about sinking from view, to see the calm, self-possessed, clear and active state of the mind in choleraic patients. That which is the rule in Asiatic cases is equally present in Louisville attacks. There is not a single sign of the disease in Asia that is not conspicuous in the seizures in Louisville. There is not the shadow of difference in the character of the attacks, whether one set are in Asia and the other are in Louisville. An attack of intermittent fever is precisely the same as an attack of that disease in Louisville. Why should one be called Bothnia fever, the other Louisville fever?

In Asia there are large regions among the places devastated with cholera that have never had a case of the disease in them. One village may be severely visited one season; another village in the immediate vicinity of it, having intimate intercourse with the afflicted one, has never had a case of disease among its inhabitants. That is precisely the history of the disease in the United States. How is it that Sundeep in the sunderbunds along the Bay of Bengal always escaped from cholera, while the ravages around it were dreadful? The escape was not due to the want of intercourse.

Kristofsky, near St. Petersburg, was perfectly free from the disease, while St. Petersburg was severely ravaged, and there was constant intercourse between the two places. Vienna suffered severely from cholera; it raged around the Faubourg Leopoldstadt at Vienna, but this Faubourg escaped. The intercourse between this and the ravaged parts of Vienna was constant.

In 1833, Lexington, Kentucky, was very severely ravaged with cholera—more than one-half the population of Lexington fled from it. They did not carry the disease nor spread it. The intercourse between Lexington and Versailles was very intimate, daily interchanges of visits took place, but there was not a case in Ver sailles.

Then, again, "The testimony is conclusive that the German villages of Gallicia have always been spared; this exemption is due to their cleanliness, but many places around these villages that were known for their insanitary condition the disease ravaged severely. The town of Sarepta, noted for its thorough cleanliness, has always escaped a visitation of cholera, while neighboring places, not conspicuous for cleanliness, suffered from its fatal ravages.

In Hindostan the natives universally noticed that villages very unhealthy and exposed to the exhalations from marshes, rivers and lakes, were sure to suffer; while other places in different conditions entirely escaped. Mr. Jameson, who gathered these facts on a large scale, had his attention called to this feature by noticing the towns of Muttra and Agra. Muttra, (often spelled Mathura) is a very filthy, crowded town. Although it was forty miles nearer the line of cholera than Agra, the latter being dry and airy, Muttra was fatally assailed while Agra was scarcely touched. Jameson says these facts are true about the whole of India. It was often noticed in India that the wives of soldiers, twenty-three out of one hundred and fifty-nine dying with cholera, while among forty-two ladies in the barracks there was not a single case. These then are the testimonies of India on this subject. Surely those who are affected with an Asiatic mania, whenever cholera is mentioned can afford to listen to these potent voices from Asia.

Before leaving Asia I beg leave to call attention to important facts connected with a celebrated spot in India. It has important features that are very instructive, and to which we cannot give too much heed. In the midst of an immense granitic plain an immense granite rock shoots up five hundred feet. Upon the summit of this rock the British determined to erect a fort, because of its commanding position. There were no springs nor streams there to supply it with water. The engineers had capacious cisterns cut in the granite, and relied upon rain-water for the garrison. The fort was called Fort Bellary. It soon became notorious for the ravages of cholera. It is the only place in the world that was known to have cholera annually. It was very fatal. The garrison was composed of infantry, cavalry and artillery, and the hill-sides were well covered with the graves of these members of the force. It was noticed that while cholera ravaged the fort annually, no case had ever occurred in the Bazar in the plain near the foot of the hill.

When McGregor became Medical Director of the British forces in India, he determined to make a personal inspection of Fort Bellary. It was high time that some one should do this. He went to the scene of these annual ravages, and speedily unveiled the mystery. He found immense masses of decaying vegetable matter about the water-tanks, where the horses were fed and watered. He ordered the immediate removal of this mass, and provided for thorough drainage so as to cut off the supply of moisture. He instituted proper measures for feeding and watering the animals, and required that due attention should be paid to daily cleanliness. From that time down to the present, through a period of over thirty years, there has never been a case of cholera at Fort Bellary. In all cases, if we change a place, that has had cholera, into the exact similitude of a place that never had a case, we acquit ourselves of a public duty under the guidance of a supreme wisdom, and we then feel that our labors are not in vain. Since Medical Director McGregor performed this duty at Fort Bellary, no mortal has ever been able to make cholera "travel" to Fort Bellary, nor to "travel" from it. McGregor struck it a deadly blow, and at Fort Bellary it has been a nonentity ever since. This was a medical duty recognized and effectively performed.

The question naturally springs up and demands an answer—Have such results followed similar labors elsewhere besides Fort Bellary? We do not know an instance to the contrary. We have been intimate with such scenes for nearly fifty-one years, and in that extensive experience we have never known a spot visited by cholera, the condition of which was so changed as to resemble places that never had a case of the disease, in which it ever appeared again. There is no more reason to apprehend that cholera can again attack Market street between Tenth & Eleventh streets, than we have to apprehend any other impossibility. No one feels any more fear that it can again attack both sides of Jefferson street, beginning at the corner of Jackson street, as it did in 1850, than that it can sweep the Galt House or Louisville Hotel clear of inhabitants. No effect can come without a cause. When a cause is absent, the effect can not appear. No one has any uneasiness that it may again show itself on the corner of Ninth and Jefferson and ravage nearly up to Eighth street, as it did in 1832. There are many hundreds of squares in Louisville that never have had a case of cholera in them. It is perfectly reasonable to feel sure that if we put every square in the city in the condition that those were in when cholera failed to attack them, we shall shut it out completely. This seems as plain to us as that twice four make eight. Many places here that had a cholera visitation in 1832 never have had a case since. Spots that had a visitation in 1833, and were changed, never have been afflicted with cholera since, nor anything akin to it down to this moment.

We shall record the triumphant labors of Dr. Shapter, at Exeter, England, and those that have saved the great lunatic asylum at

Bethlehem, England, ever since 1832. But the especial points of the inquiry on this interesting matter will be devoted to home. We prefer this, because the facts can be denied or proven most easily where all of them are best known among living witnesses. To this inquiry we shall devote the next number of this series. In the meantime we urge upon all the necessity of thorough cleanliness and dryness at home, and in all their surroundings, as the price at which health may be secured.

The question is often asked, with deep solicitude, when we hear that the disease has broken out anywhere, are we going to have cholera here? We can enable each one to answer this question for himself. Very carefully examine your premises, leave nothing uninvestigated; then thoroughly know the condition of all your surroundings, for it should be well recognized that, if some neighbor has dangerous premises, you cannot be safe. Let each one see that his own house and grounds are dry, airy, and clean; that the contents of his privy-pit are at least three feet below the surface, and in this state of things he may feel perfectly secure so far as his own premises are concerned. Then, if he finds a damp spot where vegetable material is decomposing, let him take the proper steps for removing that. All this being done, he may feel as certain that he has nothing to fear from cholera as he can feel in any earthly matter. In examining his premises he may overlook a source of great danger. The surface may look dry, but there may be water beneath it that may imperil life—if so, drain this water off.—*Louisville Med. News.*

